# Cross-Border Sexual Transmission of the Newly Emerging HIV-1 Clade CRF51_01B

**DOI:** 10.1371/journal.pone.0111236

**Published:** 2014-10-23

**Authors:** Hui Ting Cheong, Kim Tien Ng, Lai Yee Ong, Jack Bee Chook, Kok Gan Chan, Yutaka Takebe, Adeeba Kamarulzaman, Kok Keng Tee

**Affiliations:** 1 Centre of Excellence for Research in AIDS (CERiA), Department of Medicine, Faculty of Medicine, University of Malaya, Kuala Lumpur, Malaysia; 2 Division of Genetics and Molecular Biology, Institute of Biological Sciences, Faculty of Science, University of Malaya, Kuala Lumpur, Malaysia; 3 AIDS Research Center, National Institute of Infectious Diseases, Toyama, Shinjuku-ku, Tokyo, Japan; Institut Pasteur of Shanghai, Chinese Academy of Sciences, China

## Abstract

A novel HIV-1 recombinant clade (CRF51_01B) was recently identified among men who have sex with men (MSM) in Singapore. As cases of sexually transmitted HIV-1 infection increase concurrently in two socioeconomically intimate countries such as Malaysia and Singapore, cross transmission of HIV-1 between said countries is highly probable. In order to investigate the timeline for the emergence of HIV-1 CRF51_01B in Singapore and its possible introduction into Malaysia, 595 HIV-positive subjects recruited in Kuala Lumpur from 2008 to 2012 were screened. Phylogenetic relationship of 485 amplified polymerase gene sequences was determined through neighbour-joining method. Next, near-full length sequences were amplified for genomic sequences inferred to be CRF51_01B and subjected to further analysis implemented through Bayesian Markov chain Monte Carlo (MCMC) sampling and maximum likelihood methods. Based on the near full length genomes, two isolates formed a phylogenetic cluster with CRF51_01B sequences of Singapore origin, sharing identical recombination structure. Spatial and temporal information from Bayesian MCMC coalescent and maximum likelihood analysis of the protease, gp120 and gp41 genes suggest that Singapore is probably the country of origin of CRF51_01B (as early as in the mid-1990s) and featured a Malaysian who acquired the infection through heterosexual contact as host for its ancestral lineages. CRF51_01B then spread rapidly among the MSM in Singapore and Malaysia. Although the importation of CRF51_01B from Singapore to Malaysia is supported by coalescence analysis, the narrow timeframe of the transmission event indicates a closely linked epidemic. Discrepancies in the estimated divergence times suggest that CRF51_01B may have arisen through multiple recombination events from more than one parental lineage. We report the cross transmission of a novel CRF51_01B lineage between countries that involved different sexual risk groups. Understanding the cross-border transmission of HIV-1 involving sexual networks is crucial for effective intervention strategies in the region.

## Introduction

HIV-1 evolves rapidly with its error-prone replication cycles, a short generation time, and great tendency to recombine [1], [2]. Studies have shown that concurrent circulation of multiple genotypes may lead to elevation in complexity and diversity of HIV-1 molecular evolution through generation of inter-subtypes recombinant strains with significant epidemiological impact, known as the circulating recombinant forms (CRFs).

In Southeast Asia, cases of HIV-1 infection in Singapore has been on the rise for the past decade, with sexual intercourse being the major route of HIV transmission (>95%), predominantly through heterosexual and homosexual risk behaviours [3]. This coincides with the scenario in Malaysia, which saw an increase in proportion of sexually transmitted HIV-1 cases in the last few years [4]. As both countries have close socioeconomic ties, the risk for cross-border transmission of HIV-1 between Malaysia and Singapore is imminent. Previous reports showed that HIV-1 molecular epidemics in Singapore depicted a distinctive pattern of genotype discriminations in different key risks populations. In the late 1990 s, CRF01_AE and subtype B of western origin were the major HIV-1 strains circulating in Singapore, mainly introduced by the heterosexual and the homosexual risk groups, respectively [5]. More recently, although similar epidemiological trend was reported in both risk groups, approximately 12% of the viral isolates were not clustered with any known subtype or CRFs [6]. The unique cluster was later designated as CRF51_01B, a novel CRF clade generated from the two major circulating strains in Singapore - CRF01_AE and subtype B [7]. Despite the close proximity of both countries, CRF51_01B have yet to be reported in the Kuala Lumpur city in Malaysia [8].

As of late, cases of HIV-1 transmission between neighbouring countries are being reported with increased frequency, due mainly to the greater availability of viral genomic sequences. For instance in China, phylogenetic studies have shown that multiple CRF01_AE lineages circulating in the country were originated from Southeast Asia (Thailand and Vietnam) through independent transmission events [9]–[11]. Likewise, purported transmission of subtype C from India to China set off a chain of events including the generation of CRF07_BC and CRF08_BC in China, followed by the sequential migration of CRF07_BC to Taiwan among injecting drug users (IDU) [12], [13]. Cross transmission of subtype B and CRF01_AE lineages native to China and Japan were also observed among men who have sex with men (MSM) [14]. Most recently, the phylodynamic profiles and spread of a newly emerging CRF55_01B among MSM was also reported in China [15]. Such observations suggest that the molecular evolution of HIV-1 is a highly dynamic course, and that distinct epidemic in different countries would eventually converge upon each other if permitted to spread without proper intervention. Coupled with the tendency of HIV-1 to recombine, such cross transmission events would inevitably increase the overall complexity and diversity of HIV-1 in any particular country. In this study, we investigated the molecular epidemiology of HIV-1 in Singapore and Malaysia by tracing the spatiotemporal origin and possible events of transmission between risk groups and countries involving the newly discovered HIV-1 CRF51_01B.

## Materials and Methods

### Ethics Statement

The study was approved by the University Malaya Medical Centre (UMMC) Medical Ethics Committee. Standard, multilingual consent forms allowed by the Medical Ethics Committee were used. Written consent was obtained from all study participants.

### Study Subjects and Near Full Length Genomes of HIV-1 CRF51_01B

In order to investigate the geographical origin of HIV-1 CRF51_01B and its possible movement between Malaysia and Singapore, a retrospective molecular epidemiological analysis was conducted among 595 subjects recruited for an antiretroviral resistance surveillance study in the city of Kuala Lumpur, Malaysia, between 2008 and 2012. HIV-1 RNA was extracted from plasma through magnetic silica-based method implemented in the automated NucliSENS easyMAG platform (BioMerieux, France). Reverse transcription was carried out with random hexamers using SuperScript III RNase H^−^ Reverse Transcriptase (Invitrogen, Carlsbad, California, USA) according to manufacturer’s instruction. Next, sets of primers previously described [8] were used to amplify the protease and reverse transcriptase genes through nested PCR with QIAGEN HotStarTaq Plus DNA Polymerase (Qiagen, Hilden, Germany). PCR products were purified and sequencing was performed in an ABI PRISM 3730XL DNA Analyzer with BigDye terminators (Applied Biosystems, Foster City, California, USA).

In order to discern the subtypes of amplified sequences, reference sequences relevant to the Southeast Asia region including three near full length and all partial genomic sequences of CRF51_01B [6], [7] were downloaded from Los Alamos National Laboratory (LANL) HIV sequence database (http://www.hiv.lanl.gov/) for phylogenetic analysis. References and query sequences were aligned in accordance to HIV Sequence Compendium 2012 (http://www.hiv.lanl.gov/). Neighbour-joining analysis was carried out to discern the phylogenetic relationships of all the isolates in MEGA version 5 [16] with Kimura two-parameter model with a transition-transversion ratio of 2.0. Amplification of the complete genomic sequence for isolates that formed a monophyletic cluster with known CRF51_01B was carried out using primers as described elsewhere [17]. All CRF51_01B sequences reported in this study, 11MYKL055 and 09MYKL050 have been deposited in GenBank with accession numbers KJ485697 and KJ485698, respectively.

### Recombination and Phylogenetic Analysis

Recombinant Identification Program (RIP) tool from LANL HIV sequence database was used to depict recombination structure in submitted query sequences through comparison with a background alignment [18]. Subsequently, bootscanning and informative sites estimation [19] using SimPlot version 3.5.1 [20] were carried out to determine the mosaic structures of the recombinants as well as the location of breakpoints. Next, sub-region neighbor-joining trees were built for each recombinant segment to reaffirm the identity or parental origin of the genomic fragments.

In order to improve resolution of subsequent phylogenetic analysis, additional genomic sequences of subtype B and CRF01_AE from Singapore and Malaysia were downloaded from LANL HIV sequence database and GenBank database. These sequences were then aligned against reported CRF51_01B strains as well as other reference sequences, and neighbour-joining analysis was carried out. The criteria for confirming partial sequences to be CRF51_01B isolates is that sequences must be available in all three genomic regions of protease (subtype B), gp120 (CRF01_AE) and gp41 (subtype B), and these sequences should cluster with known/reported CRF51_01B with strong bootstrap support. This also served as a preliminary analysis to identify subtype B and CRF01_AE strains that are closely related to CRF51_01B in protease, gp120 and gp41 genomic regions.

Maximum likelihood analysis for the Malaysian CRF51_01B sequences was performed using PAUP version 4.0 [21] on the protease, gp120 and gp41 regions along with sequences of known Singaporean CRF51_01B isolates downloaded from the LANL HIV sequence database. In order to deduce the timeline for emergence of CRF51_01B isolates in Singapore and Malaysia, Bayesian Markov chain Monte Carlo (MCMC) sampling method was performed to obtain the posterior distribution of phylogenies in BEAST version 1.7.5 [22] under the uncorrelated log-normal relaxed clock model [23] with general time-reversible (GTR) nucleotide substitution and constant tree models. Three independent MCMC chain runs of 30 million steps sampled for every 30000 states were performed on specific genomic regions mentioned as before. The MCMC output was checked for convergence and effective sampling size (ESS) using Tracer version 1.4 (http://tree.bio.ed.ac.uk) with 10% of each chain discarded as burn-in.

## Results

Molecular epidemiology investigation was conducted using HIV-1 sequence data from Kuala Lumpur, Malaysia and Singapore to study the early evolutionary events associated with the emergence of CRF51_01B and its possible cross-transmission between two countries. From 595 consented HIV-positive patients recruited in Kuala Lumpur between 2008 and 2012, 485 protease-reverse transcriptase genes (HXB2∶2253–3271, 1019 bp) were successfully amplified and sequenced. Out of 485 screened patients, 139, 69, 5 and 31 patients reported heterosexual, homosexual, bisexual and injecting-drug use risk behaviour, respectively. There were two groups who reported dual-risk behaviours of heterosexual with injecting drug use and homosexual with injecting drug use, each with 12 and 3 patients. Twelve pediatric subjects were also included. A remainder of 214 patients did not provide risk information.

**Figure 1:**
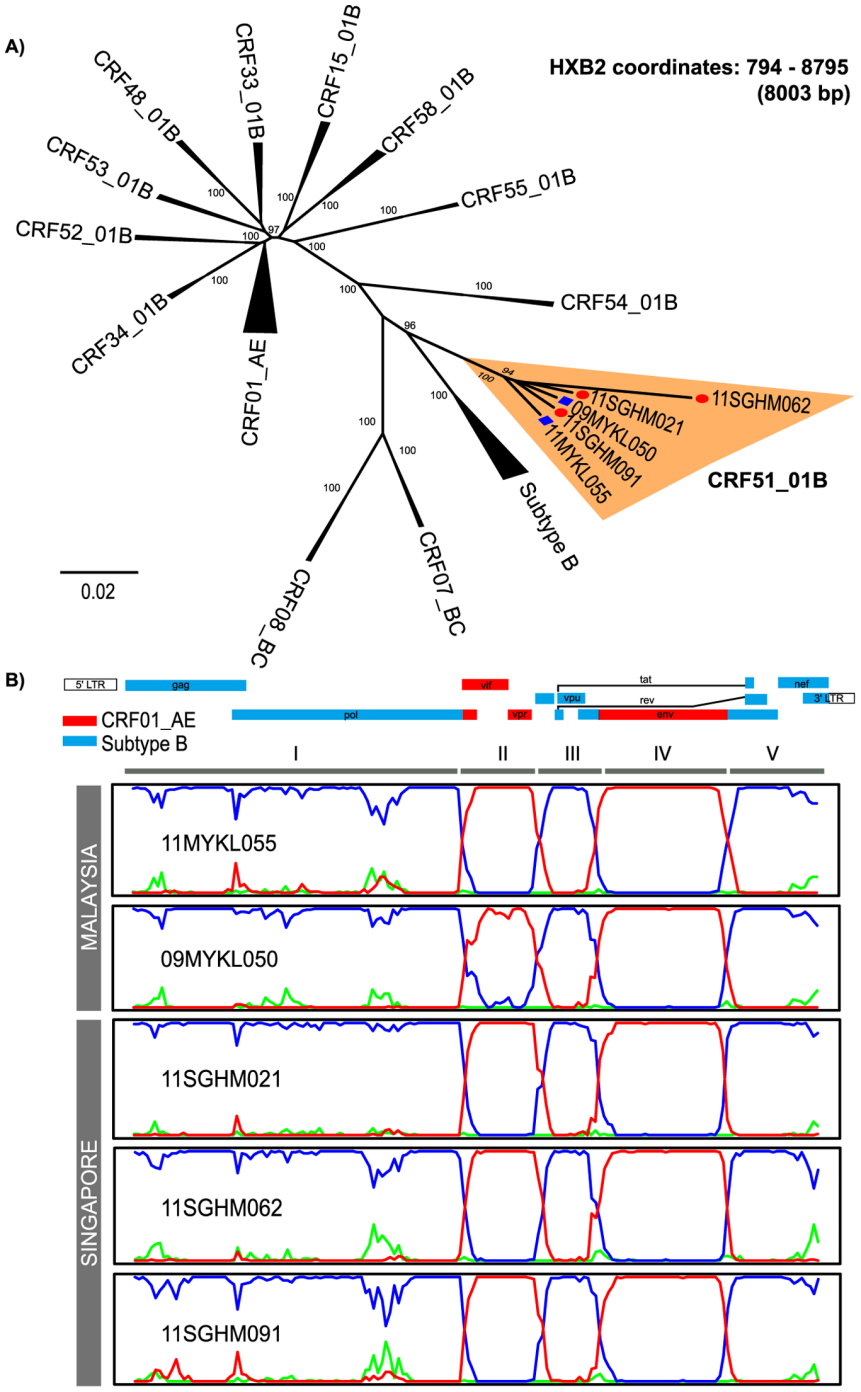
Phylogenetic and recombination analysis of HIV-1 CRF51_01B near full length genomes. **A**. Neighbour-joining analysis of the near full length sequences of 11MYKL055 and 09MYKL050 (HXB2 coordinates: 794–8795, 8003 bp) isolated from Malaysia against reference sequences of different subtypes/CRFs. CRF51_01B isolates from Singapore and Malaysia are indicated by red circles and blue squares, respectively. **B**. Bootscan analysis of all available near-full length genomes of CRF51_01B from Malaysia (11MYKL055 and 09MYKL050) and Singapore (11SGHM021, 11SGHM062 and 11SGHM091) using 83USSF2 (subtype B) of western origin, 90THCM235 (CRF01_AE) as putative parental strains, and subtype C (95IN21068) as an outlier strain. Subtype B, CRF01_AE and subtype C strains are represented by blue, red and green lines, respectively.

From the neighbour-joining analysis, more than half of the isolates were genotyped as CRF01_AE and CRF33_01B, with 215 (44.3%) and 157 (32.4%) isolates, respectively **(Figure S1)**. Two distinct lineages of subtype B were almost proportionate, with 44 (9.1%) being subtype B of Western origin and 31 (6.4%) being subtype B of Thai origin. Seventeen isolates (3.5%) were assigned as unique recombinant forms (URFs) of CRF01/B (13 isolates), subtype B/D (1 isolate), subtype B/C (1 isolate), subtype A/C (1 isolate) and subtype CRF01/B/C (1 isolate). Remaining isolates were typed as subtype C (5), CRF54_01B (4), CRF53_01B (3) CRF51_01B (2) subtype D (2), subtype G (2), CRF52_01B (1), CRF02_AG (1) and CRF48_01B (1). Two isolates, 11MYKL055 and 09MYKL050, formed a monophyletic cluster with CRF51_01B, a recombinant genotype that consists of subtype B and CRF01_AE. The reported modes of disease acquisition for subjects 11MYKL055 and 09MYKL050 were heterosexual and MSM, respectively. Near full length genomes of 11MYKL055 (HXB2∶707−9571, 8857 bp) and 09MYKL050 (HXB2∶790−9552, 8766 bp) were then amplified, sequenced and subjected to neighbour-joining analysis, thus further confirming their subtype and phylogenetic relationship (**Figure 1A**).

Distance analysis by RIP in the LANL HIV sequence database suggested the presence of a recombinant resembled that of CRF51_01B. Bootscan and informative site analysis, using 83USSF2 (subtype B), 90THCM235 (CRF01_AE) as putative parental strains and 95IN21068 (subtype C) as an outlier strain, estimated the precise recombination structure of 11MYKL055 and 09MYKL050. Five sub-regions comprising of subtype B of Western origin and CRF01_AE in the genome sequence were determined: region I (HXB2∶794−4952), III (HXB2∶5887−6428) and V (HXB2∶7829−9417) from subtype B of Western origin; region II (HXB2∶4997−5839) and IV (HXB2∶6477−7782) from CRF01_AE (**Figure 1B**). The recombination structures of 11MYKL055 and 09MYKL050 were similar to the Singaporean CRF51_01B isolates first reported in 2011 [7]. The four recombination breakpoints of CRF51_01B were located at HXB2 coordinates of 4953–4996, 5840–5886, 6429–6476 and 7783–7828.

**Figure 2:**
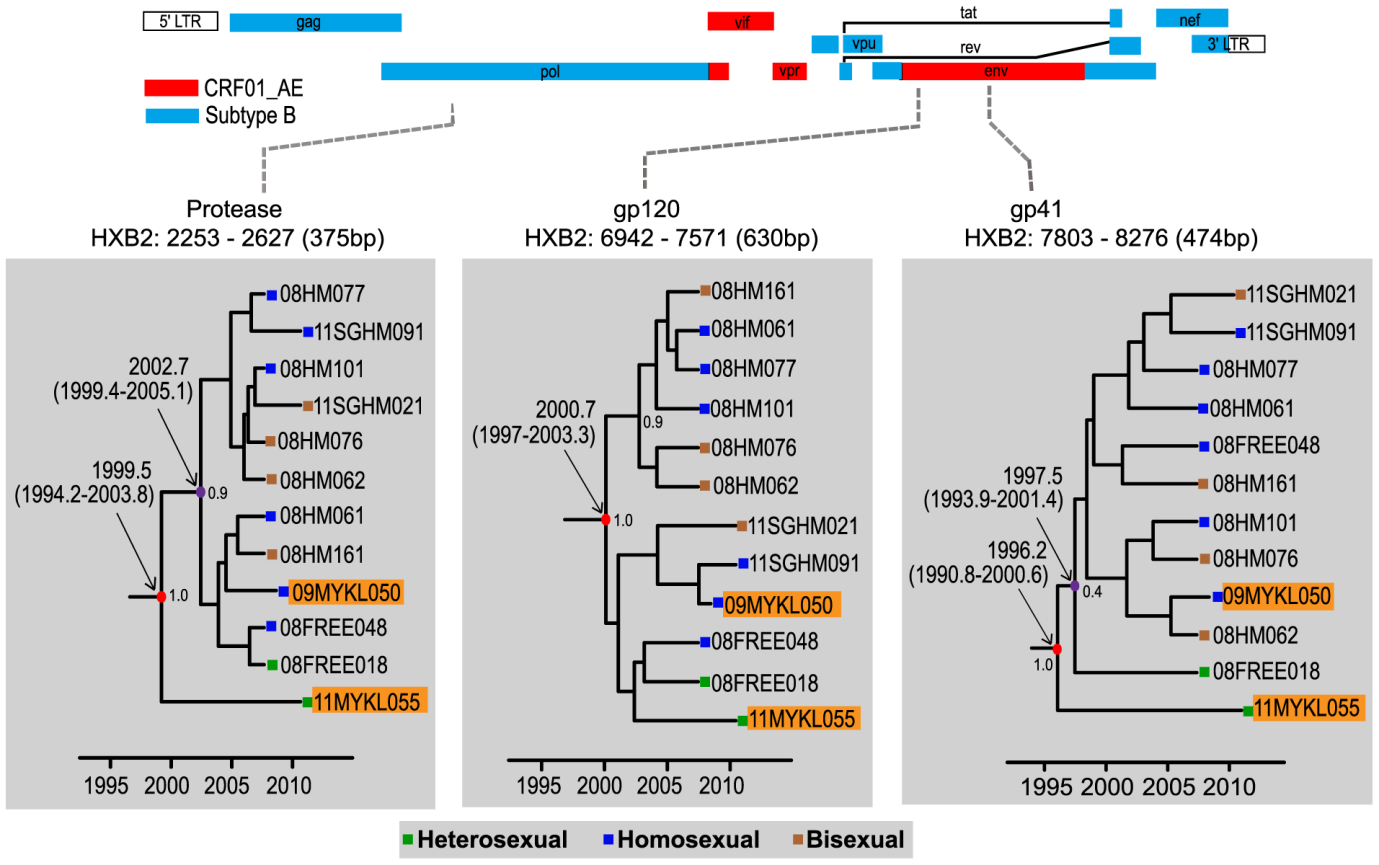
Maximum clade credibility (MCC) trees reconstruction of CRF51_01B isolated in Singapore and Malaysia. MCC trees for protease (subtype B, HXB2∶2253–2627, 375 bp), gp120 (CRF01_AE, HXB2∶6942–7571, 630 bp) and gp41 regions (subtype B, HXB2∶7803–8276, 474 bp) are shown. Timescale is shown at the bottom of the tree. The mean tMRCA and 95% highest probability density (HPD) for the key nodes are indicated. CRF51_01B strains from Malaysia are highlighted in orange.

Partial sequences from Singapore and Malaysia that clustered with CRF51_01B with significant bootstrap support were classified as CRF51_01B, provided that the protease (subtype B), gp120 (CRF01_AE) and gp41 (subtype B) genomic regions were all present. In total, twelve protease, gp120 and gp41 sequences of CRF51_01B were subjected to further analyses. Maintaining the longest lengths possible for these sequences, maximum clade credibility (MCC) and maximum likelihood tree reconstructions were carried out through the protease (subtype B, HXB2∶2253–2627, 375 bp), gp120 (CRF01_AE, HXB2∶6942–7571, 630 bp) and gp41 regions (subtype B, HXB2∶7803–8276, 474 bp), respectively. The analysis showed that 11MYKL055 consistently occupied the basal position of the CRF51_01B clade in the MCC trees of the protease and gp41 regions, but in gp120 it was intermingled with other isolates of CRF51_01B (**Figure 2**). Maximum likelihood analysis also illustrated similar tree topology (**Figure S2**). Diverging immediately from the ancestral lineage, 11MYKL055 (in the protease and gp41 genomic regions), was a group of CRF51_01B isolates from Singapore (majority MSM) that also include a Malaysian MSM, 09MYKL050. In addition, closely related subtype B and CRF01_AE sequences of Singapore origin were located directly outside the CRF51_01B clade across all genomic regions in maximum likelihood analysis.

In order to estimate the temporal origin of CRF51_01B and its possible spread between countries and/or risk groups, the protease, gp120 and gp41 alignment data sets were categorized into two separate taxa: taxon 1 contains all twelve CRF51_01B isolates, and taxon 2 contains eleven isolates, excluding isolate 11MYKL055 from Malaysia. Bayesian MCMC analysis using the uncorrelated lognormal relaxed clock model estimated the evolutionary rates for the protease, gp120 and gp41 genes at 2.3 (95% highest posterior density (HPD): 2.0–2.5) × 10^−3^, 4.3 (3.8−4.9) × 10^−3^, and 3.9 (3.6−4.1) × 10^−3^ substitutions/site/year, respectively. The time (in calendar year) of the most recent common ancestor (tMRCA) of taxon 1 in the protease, gp120 and gp41 regions were estimated at 1999.5 (95% HPD: 1994.2–2003.8), 2000.7 (1997−2003.3) and 1996.2 (1990.8−2000.6) respectively, which are essentially similar to those reported elsewhere [24]. On the other hand, the tMRCA of taxon 2 in similar genetic regions were estimated at 2002.7 (1999.4−2005.1), 2000.7 (1997.5−2003.9) and 1997.5 (1993.9−2001.4). Hence, the tMRCA estimates of taxon 2 are consistently younger than that of taxon 1 in the protease and gp41 regions while the estimates in gp120 regions for both taxa overlapped (**Table 1**). Of note, tMRCA estimates carried out for ancestral strains of subtype B' and CRF01_AE were similar to previously published data [10], [25].

**Table 1:** Evolutionary characteristics of HIV-1 CRF51_01B isolates from Singapore and Malaysia.

Genomic region	Rates of evolution^a^	tMRCA^b^	
		Taxon 1^c^	Taxon 2^d^
Protease (HXB2∶2253–2627, 375 bp)	2.3 (2.0−2.5)	1999.5 (1994.2–2003.8)	2002.7 (1999.4–2005.1)
gp120 (HXB2∶6942–7571, 630 bp)	4.3 (3.8−4.9)	2000.7 (1997.0–2003.3)	2000.7 (1997.5–2003.9)
gp41 (HXB2∶7803–8276, 474 bp)	3.9 (3.6−4.1)	1996.2 (1990.8–2000.6)	1997.5 (1993.9–2001.4)

a Estimated mean rates of evolution expressed as 10^−3^ nucleotide substitutions/site/year for the protease (subtype B), gp120 (CRF01_AE) and gp41 regions (subtype B) under a relaxed molecular clock with GTR+Γ^4^ substitution model and a constant size coalescent model. The 95% highest posterior density (HPD) confidence intervals are included in parentheses.

b Mean time of the most common ancestor (tMRCA, in calendar year) for protease (subtype B), gp120 (CRF01_AE) and gp41 (subtype B). The 95% HPD confidence intervals are indicated.

c Refers to all CRF51_01B isolates from Singapore and Malaysia.

d Refers to all CRF51_01B isolates from Singapore and Malaysia diverging from the ancestral strain 11MYKL055 who was a heterosexual.

## Discussion

To our knowledge, CRF51_01B has only been found in Singapore before now. In this study, we report the first detection of two CRF51_01B strains in Kuala Lumpur, Malaysia. Based on phylogenetic reconstructions of the partial genomes using the Bayesian MCMC and maximum likelihood methods, one of these strains (11MYKL055) appeared to be ancestral to the other isolates of CRF51_01B. Besides that, the tMRCA analysis estimated that isolate 11MYKL055 is slightly older than Singaporean isolates and the other Malaysian isolate, 09MYKL050. With that said, we proposed two possible directions of transmission of CRF51_01B: 1) CRF51_01B originated in Malaysia and subsequently transmitted to Singapore, shortly after approximately one year of its emergence; 2) CRF51_01B originated in Singapore among Malaysian residing in or travelled to Singapore before it was spread to other areas.

Though CRF51_01B was first described in Singapore, there is a high probability that it could be native to Malaysia. As one of five countries with mega-epidemics of HIV-1, this proposition is supported by the number of CRFs discovered in Malaysia in recent years such as CRF33_01B [26], CRF48_01B [27], CRF52_01B [28], CRF53_01B [29], CRF54_01B [30] and CRF58_01B [17]. Moreover, the ancestral lineage 11MYKL055 was isolated from a Malaysian. However, maximum likelihood and MCC tree reconstructions of additional genomic sequences from Singapore and Malaysia along with known CRF51_01B indicated that CRF51_01B is closely related to subtype B and CRF01_AE strains of Singaporean origin **(Figure S2 and S3)**. Through further investigations, we found that both the Malaysian patients, particularly 11MYKL055, travelled to and stayed (2–3 months per trip, multiple trips per year) in Singapore frequently in the early 2000 s, close to the estimated timeframe (calendar year 1996 to 2000) for the emergence of CRF51_01B, and his HIV-positive status was confirmed in 2006. Moreover, no further transmissions or outbreaks of CRF51_01B were detected in Malaysia besides the two isolates reported in this study, 11MYKL055 and 09MYKL050.

**Figure 3:**
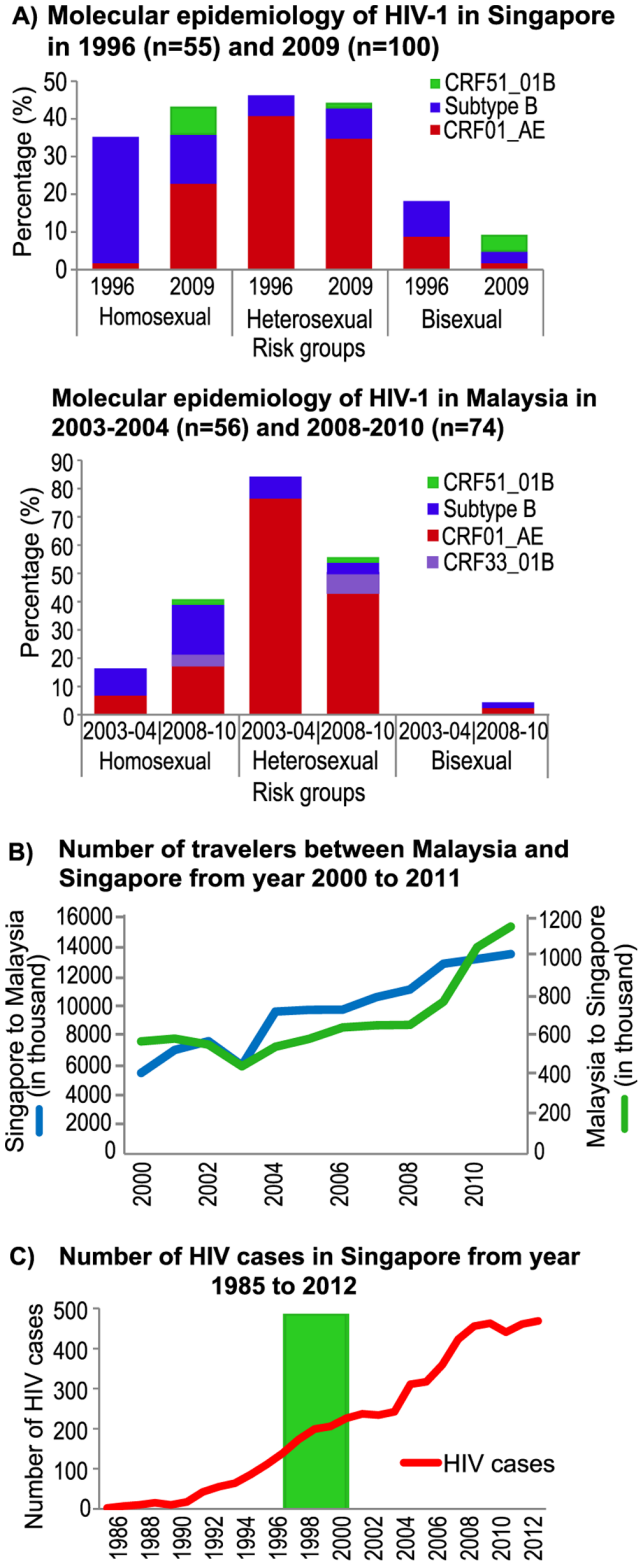
HIV-1 epidemiology and traveling trend in Singapore and Malaysia. **A**. The molecular epidemiological pattern of HIV-1 in Singapore between 1996 and 2009 are delineated in the bar chart. A significant shift from majority subtype B to CRF01_AE was evident among the homosexual group, while the subtype distribution in the heterosexual risk group remained relatively stable between 1996 and 2009. New detection of CRF51_01B was observed across all sexual risk groups in 2009. On the other hand, subtype distribution patterns in Malaysia between 2003–2004 and 2008–2010 remained relatively stable. Similar distribution trends can be observed between Singapore and Malaysia for the heterosexual populations, which were dominated by CRF01_AE. Notably, HIV-1 subtype distribution in the 2000 s was similar for the homosexual population in both countries, with CRF01_AE and subtype B in equal proportions [8], [42]. **B**. Number of travelers between Malaysia and Singapore between 2000 and 2011 [36], [43]. The close socioeconomic relations between Singapore and Malaysia can be reflected by the increasing number of travelers transiting to or from these two countries over the recent decade. **C**. Number of reported HIV-1 infections in Singapore between 1985 and 2012 [3]. The line chart depicted the increasing trend of HIV-1 infections in Singapore over a time span of 27 years. The estimated time of the most recent common ancestor (tMRCA) for CRF51_01B between 1996 and 2000 is also indicated.

Taken together, our inference inclined towards the second hypothesis, which supports Singapore as the geographical origin of CRF51_01B, but features Malaysian travelers as likely hosts in the earlier transmission events of CRF51_01B in Singapore. Since the host of 11MYKL055 was a heterosexual individual, it is possible that CRF51_01B first emerged within the heterosexual population before it was transmitted to the homosexual population within a limited period of time, based on the tMRCA estimates. However, the position of 11MYKL055 in the MCC tree for gp120 genomic region does not support this notion as said isolate was intermingled with other Singaporean isolates. This may be due to the differences in evolutionary history and origins of the distinct lineages of subtype B (Western origin) and CRF01_AE (Thai origin), as observed in this study and elsewhere [24]. Moreover, genealogical-based estimations on the HIV-1 genome are inevitably affected by recombination between lineages of the same subtype [31]–[33]. Such recombination events have been reported to cause biases in genealogical characteristics and loss of phylogenetic correlation in corresponding genomic sites, both of which could possibly lead to quantitative discrepancies [34], [35]. Based on the overlapping emergence time of the most recent common ancestor for both Malaysian and Singaporean CRF51_01B strains, there is also the possibility that 11MYKL055 was infected by an ancestral strain of CRF51_01B originated among Singaporeans. In fact, molecular epidemiological data from Singapore and Malaysia showed that CRF01_AE and subtype B co-circulated in equal proportions in the homosexual population in both countries (**Figure 3A**), which could provide a favourable environment for recombination of these subtypes, and also explain why majority of the CRF51_01B infections were found among homosexual individuals. Therefore, the homosexual population is the more likely host population for the origination of CRF51_01B. In any case, the transmission of CRF51_01B between Malaysian and Singaporean occurred in a narrow time frame, which might indicate an intricate transmission network between these two countries.

Including both isolates of Malaysian origin described in this study, it is possible to speculate that CRF51_01B is circulating exclusively among the high-risk populations involving sexual contact such as heterosexual, homosexual and bisexual risk groups at the moment. More importantly, its emergence signifies the increasing complexity of HIV-1 epidemics among these populations at risks, particular within the homosexual risk group (mostly MSM), as the shift in epidemiological pattern (i.e. higher number of CRF51_01B) is most prominent among them (**Figure 3A**). On top of that, an apparent lag in time between the estimated emergence and first report of CRF51_01B in 2011 was also noted. This showed that CRF51_01B was probably not a recently emerged CRF, as tMRCA placed its emergence to be approximately between 1996 and 2000. The discrepancies in estimated age of subtype B in protease and gp41 regions suggested that CRF51_01B may have arisen through multiple recombination events from more than one parental lineage.

The directionality of CRF51_01B transmission from Singapore to Malaysia was evident in this study despite the low detection rate of CRF51_01B in Kuala Lumpur, which is located more than 300 km from Singapore. A major factor that may increase the spread of CRF51_01B is the fact that Malaysia and Singapore are socio-economically intimate as neighbouring countries. In year 2011 alone, the number of travelers from Singapore to Malaysia was twice the total recorded in year 2000 [36] (**Figure 3B**). Since potential bridges exist for HIV transmission between communities of heterosexual, homosexual individuals and the general population [37], the possibility for cross border transmission of CRF51_01B is high as the number of HIV-1 cases in Singapore increases over the last decade [3] (**Figure 3C**).

Though statistics on the proportion of HIV-1 infections attributable to travel are not readily available, the correlation between travel and sexually transmitted diseases (STDs) such as HIV-1 should not be taken lightly, as was observed in this study as well as in others [38], [39]. Besides that, most STDs have been known to have longer incubation period and delayed diagnosis is common, travelers could be carriers themselves or unknowingly become infected and cause onward transmission of pathogens [40], [41]. Therefore, sexual transmission networks in both Malaysia and Singapore are important priorities when designing intervention strategies. Molecular epidemiological surveillance of HIV-1 between these two countries should be conducted in a regular and timely manner in order to detect possible occurrence of transnational transmission in the future.

With that, several limitations in this study should also be addressed. For one, the sampling of HIV-1 positive patient subjects for the 485 amplified PRRT sequences was conducted in Kuala Lumpur alone, which may explain the overall low prevalence of CRF51_01B in Malaysia. As further transmission of CRF51_01B within Malaysia remains unclear, the extent of CRF51_01B spread in the region could be underestimated until more studies are done in other major cities in Malaysia. Besides that, the resolution of the analyses conducted in this study might have been constricted by different molecular screening methodology between Singapore and Malaysia (eg. difference in targeted genomic regions).

All in all, phylogenetic and coalescent analyses conducted in this study provided evidence that Malaysian residing in or travelled to Singapore were among the earliest to be infected by CRF51_01B before it was spread to other Singaporeans within a narrow timeframe (within a year) and most prominently among homosexual individuals. The risk for HIV-1 spread between Singapore and Malaysia is imminent and therefore steps should be taken to prevent further transmission.

## Supporting Information

Figure S1 **HIV-1 subtype distribution for 485 patients recruited in Kuala Lumpur, Malaysia between year 2008 and 2012.** Details are included in in-figure legend.
(PDF)

Figure S2 **Maximum likelihood analysis of the HIV-1 protease, gp120 and gp41 genes of CRF51_01B.** CRF51_01B strains from Malaysia are indicated in yellow.
(PDF)

Figure S3 **Maximum clade credibility (MCC) tree reconstructions between CRF51_01B, subtype B and CRF01_AE strains from Malaysia and Singapore in the protease, gp120 and gp41 genomic regions.**
(PDF)
