# Supplementary figures and images for: Cross-Border Sexual Transmission of the Newly Emerging HIV-1 Clade CRF51_01B
Source: PLoS One. 2014 Oct 23;9(10):e111236. doi: 10.1371/journal.pone.0111236 (PMC4207770; doi:10.1371/journal.pone.0111236)

HXB2: 2253 - 3271 (1019bp)

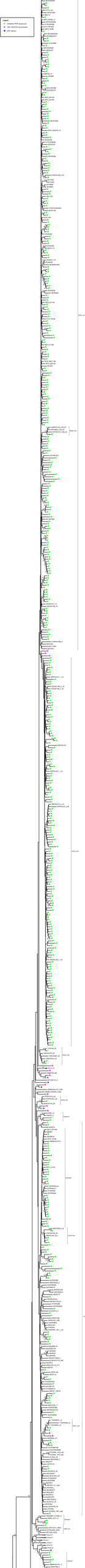

Supplement: Figure S1 — HIV-1 subtype distribution for 485 patients recruited in Kuala Lumpur, Malaysia between year 2008 and 2012. Details are included in in-figure legend. (PDF) [file pone.0111236.s001.pdf]

Figure S2

**Protease**

HXB2: 2253 - 2627 (375bp)

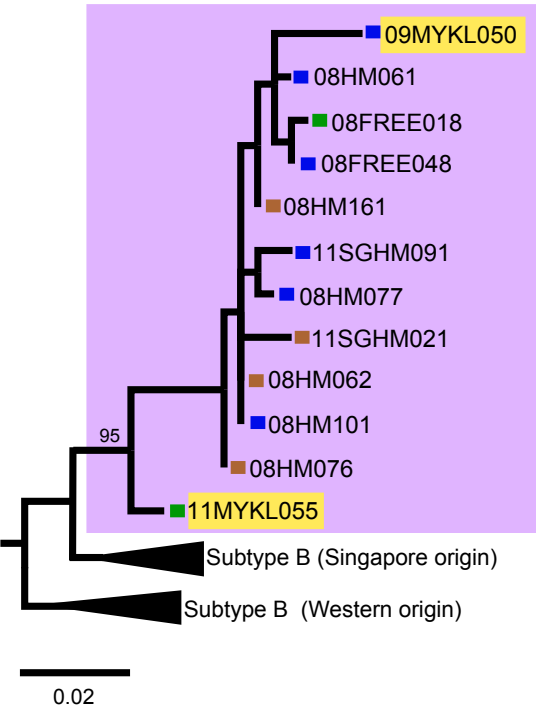

**gp120**

HXB2: 6942 - 7571 (630bp)

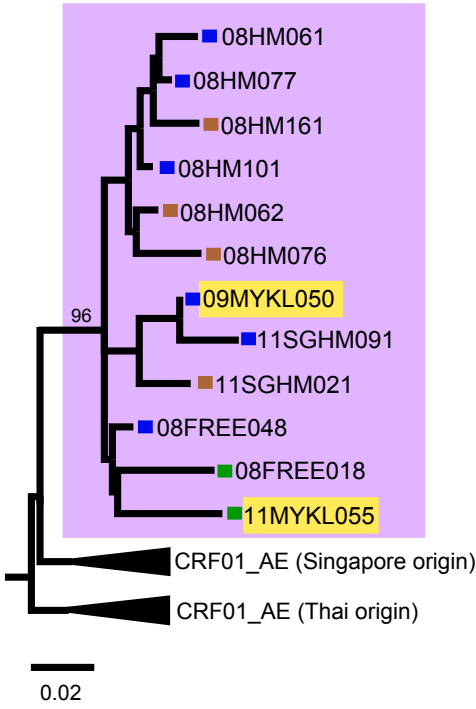

**gp41**

HXB2: 7803 - 8276 (474bp)

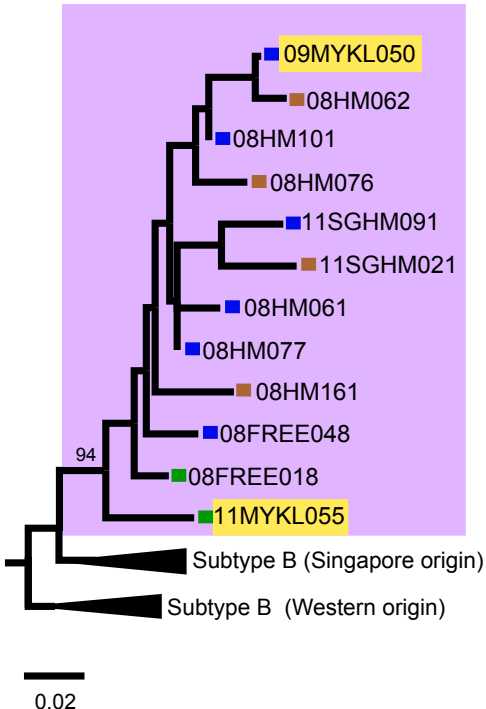

■ Heterosexual ■ Homosexual ■ Bisexual

Supplement: Figure S2 — Maximum likelihood analysis of the HIV-1 protease, gp120 and gp41 genes of CRF51_01B. CRF51_01B strains from Malaysia are indicated in yellow. (PDF) [file pone.0111236.s002.pdf]
