# Supplementary material for: Cross-Border Sexual Transmission of the Newly Emerging HIV-1 Clade CRF51_01B
Source: PLoS One. 2014 Oct 23;9(10):e111236. doi: 10.1371/journal.pone.0111236 (PMC4207770; doi:10.1371/journal.pone.0111236)

Figure S3

Protease (HXB2: 2253-2627; 375bp)_Subtype B

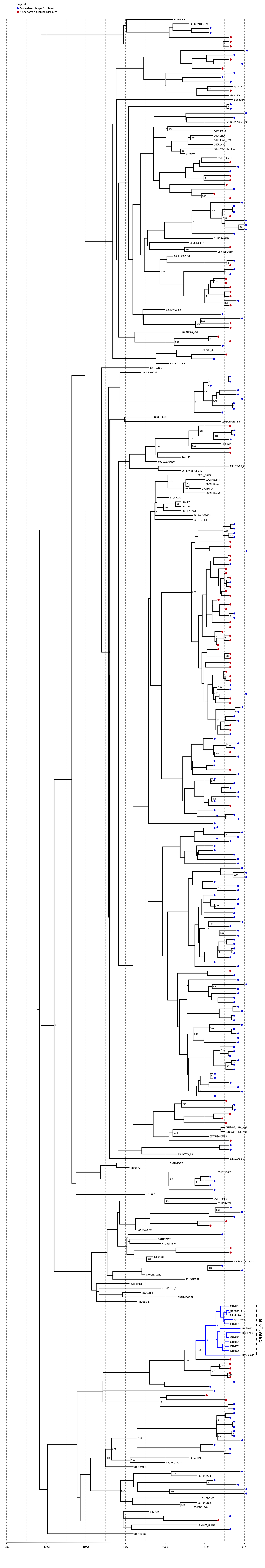

gp120 (HXB2: 6942-7571; 630bp)_CRF01_AE

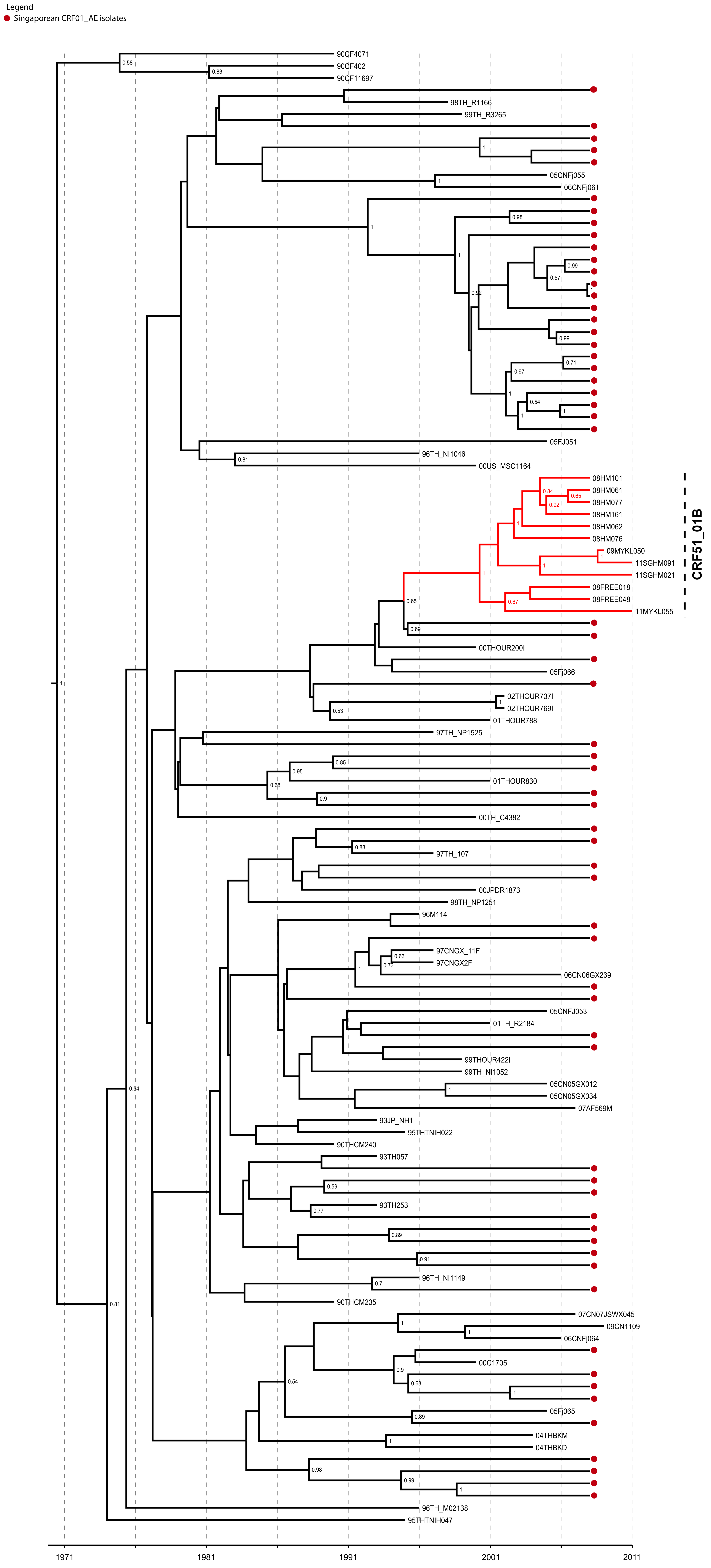

gp41 (HXB2: 7803-8276; 474bp)_Subtype B

Legend
● Singaporean subtype B isolates

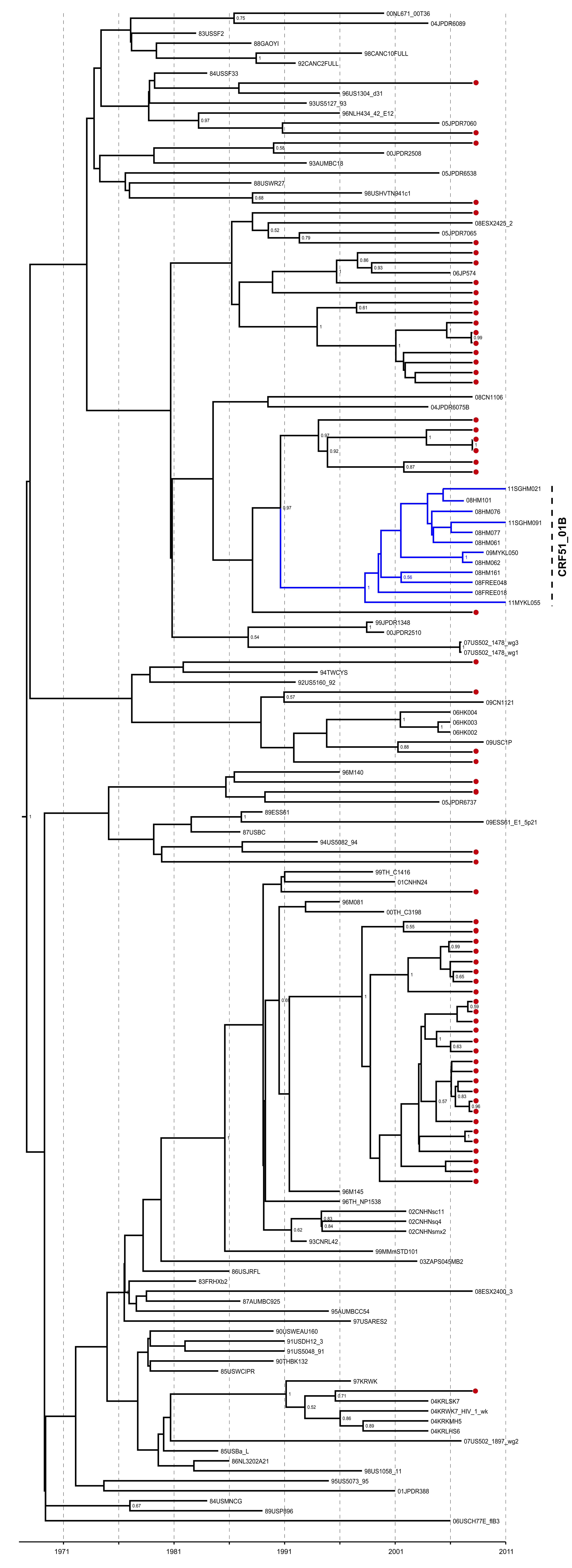

Supplement: Figure S3 — Maximum clade credibility (MCC) tree reconstructions between CRF51_01B, subtype B and CRF01_AE strains from Malaysia and Singapore in the protease, gp120 and gp41 genomic regions. (PDF) [file pone.0111236.s003.pdf]
